# Supplementary material for: Loss of growth differentiation factor 9 causes an arrest of early folliculogenesis in zebrafish–A novel insight into its action mechanism
Source: PLoS Genet. 2022 Dec 15;18(12):e1010318. doi: 10.1371/journal.pgen.1010318 (PMC9799306; doi:10.1371/journal.pgen.1010318)
Supplement: S1 Table — (DOCX) [file pgen.1010318.s001.docx]

**S1 Table. Primer list**

| Gene symbol | Sequence (5’-3’) | Application |
| --- | --- | --- |
| *gdf9* | Forward: GTTACCTACCCAGATCATTA | Mutation check |
|  | Reverse: CTCCTACAGTTTGAATGCTGCA |  |
|  | Forward: ATTATGGCGACGCTGTTTTT | HRMA |
|  | Reverse: CGTTTTCAAAGTTGTAGCTTGATG |  |
| *ef1a* | Forward: GGCTGACTGTGCTGTGCTGATTG | qPCR |
|  | Reverse: CTTGTCGGTGGGACGGCTAGG |  |
| *fshr* | Forward: CAAGACCTCACCTGAACAACAGCAGC | qPCR |
|  | Reverse: GGTCGGCGAAAGCCAAGTGGCACAT |  |
| *lhcgr* | Forward: GACGGCCTGAAAGGAGTAAG | qPCR |
|  | Reverse: GCGCAGATTCAGGTTATCAC |  |
| *cyp19a1a* | Forward: TCAGGACAATGCGTGTGGAG | qPCR |
|  | Reverse: GAGAGTTTGTGGAGGTGGTG |  |
| *inha* | Forward: GAGCCTCCTCTGCCAGTGTT | qPCR |
|  | Reverse: ATGTTGATGGAAGCGATGGTCTC |  |
| *inhbb* | Forward: TAGGGAGGACGGCAGGGTTG | qPCR |
|  | Reverse: TCGTTGGAGATCAGAAAGTAGAGGC |  |
| *inhbaa* | Forward: AACAGGCAGAACAGACGGAGATC | qPCR |
|  | Reverse: GCAGCCGAATGTTGACGTTAGC |  |
| *inhbab* | Forward: AGCCCTTCGAGATCATCACCTTC | qPCR |
|  | Reverse: GCCTGCTCCACCACTGACAG |  |
| *egfr* | Forward: GACGACCGCATGCATTTACC | qPCR |
|  | Reverse: TTCAGGCTCACAGAGTGCAG |  |
